# Prebiotic Supplementation During Gestation Induces a Tolerogenic Environment and a Protective Microbiota in Offspring Mitigating Food Allergy

**DOI:** 10.3389/fimmu.2021.745535

**Published:** 2022-01-05

**Authors:** Amandine Selle, Carole Brosseau, Wieneke Dijk, Angéline Duval, Grégory Bouchaud, Anais Rousseaux, Aurélia Bruneau, Claire Cherbuy, Mahendra Mariadassou, Véronique Cariou, Sebastien Barbarot, Marie Bodinier

**Affiliations:** ^1^ Institut National de Recherche pour l’Agriculture, l’alimentation et l’Environnement (INRAE), UR1268 Biopolymères Interactions Assemblages (BIA), Nantes, France; ^2^ Micalis Institute, Institut National de Recherche pour l’Agriculture, l’alimentation et l’Environnement (INRAE), AgroParisTech, Université Paris-Saclay, Jouy-en-Josas, France; ^3^ MaIAGE, UR1404, Institut National de Recherche pour l’Agriculture, l’alimentation et l’Environnement (INRAE), Jouy-en-Josas, France; ^4^ StatSC, École nationale vétérinaire, agroalimentaire et de l'alimentation de Nantes-Atlantique (ONIRIS), Institut National de Recherche pour l’Agriculture, l’alimentation et l’Environnement (INRAE), Nantes, France; ^5^ Department of Dermatology, Centre Hospitalier Universitaire (CHU) Nantes, Nantes, France; ^6^ Institut National de Recherche pour l’Agriculture, l’alimentation et l’Environnement (INRAE) Pays de la Loire, UMR1280 PhAN, Nantes, France

**Keywords:** food allergy, gut microbiota, immune tolerance, prebiotics, pregnancy

## Abstract

Food allergy is associated with alterations in the gut microbiota, epithelial barrier, and immune tolerance. These dysfunctions are observed within the first months of life, indicating that early intervention is crucial for disease prevention. Preventive nutritional strategies with prebiotics are an attractive option, as prebiotics such as galacto-oligosaccharides and inulin can promote tolerance, epithelial barrier reinforcement, and gut microbiota modulation. Nonetheless, the ideal period for intervention remains unknown. Here, we investigated whether galacto-oligosaccharide/inulin supplementation during gestation could protect offspring from wheat allergy development in BALB/cJRj mice. We demonstrated that gestational prebiotic supplementation promoted the presence of beneficial strains in the fecal microbiota of dams during gestation and partially during mid-lactation. This specific microbiota was transferred to their offspring and maintained to adulthood. The presence of B and T regulatory immune cell subsets was also increased in the lymph nodes of offspring born from supplemented mothers, suggestive of a more tolerogenic immune environment. Indeed, antenatal prebiotic supplementation reduced the development of wheat allergy symptoms in offspring. Our study thus demonstrates that prebiotic supplementation during pregnancy induces, in the offspring, a tolerogenic environment and a microbial imprint that mitigates food allergy development.

## Introduction

Food allergy (FA) is a common non-communicable disease that affects up to 10% of infants in some countries and has been increasing in prevalence over the last few decades (1). FA, whose symptoms can be observed in the first months of life, is linked to the dysregulation of the three key factors: the immune system (IS), the commensal microbiota, and the epithelial barriers.

Indeed, FA is characterized by a failure of oral tolerance induction and by a predominantly T helper (Th) 2-skewed immune dysregulation, regulatory T cells (Treg) dysfunction, and the secretion of antigen-specific immunoglobulin (Ig) E and IgG1 (2, 3). During allergen-specific immunotherapy, CD4^+^Foxp3^+^ Treg development is essential for the establishment of mucosal tolerance suppressing interleukin (IL)-4 production (4–6). Furthermore CD4^+^Foxp3^+^ Treg and CD8^+^Foxp3^+^ Treg cooperate to limit allergic symptoms *via* IL-10 secretion during allergen immunotherapy (7). Regulatory B cells (Breg) regulate immune responses by suppressing effector T cells through the production of anti-inflammatory cytokines (IL-10 and TGF-β) (8). In mice, IL-10-secreting Breg decrease anaphylaxis following a challenge with cow’s milk allergens through the induction of CD4^+^Foxp3^+^ Treg (9).

In parallel to oral tolerance failure, studies have measured microbial diversity and composition in populations with and without FA and have provided direct evidence that the gut microbiota of individuals with FA are different and contain specific genera (10). The absence of FA was associated with an increased abundance of *Bifidobacterium*, *Faecalibacterium prausnitzii*, and *Akkermansia muciniphila*; in contrast, a decreased abundance of *Escherichia coli* was associated with FA (11). Furthermore, bacterial therapy with *Clostridiales* or Bacteroidales species, which are deficient in young FA patients, suppressed FA in a mouse model, indicating that the loss of protective species is a key characteristic of FA disease (12). Both the IS and the gut microbiota are shaped during the first 1,000 days of life. Indeed, the early gut microbiota is crucial for the development of oral tolerance (13), and the alteration of the gut microbiota in early life can cause immune effects that can persist into adulthood and increase susceptibility to allergy (14).

FA may in part be explained by the Developmental Origins of Health and Disease (DOHaD) concept that links an individual’s health to its environmental exposure during fetal development and infancy (15). Among environmental factors, food (processed products, additives, and high fiber) plays a pivotal role in triggering the onset of FA by impacting microbiota diversity and richness and IS maturation. In this context, the maternal diet is important in shaping the offspring’s microbiome and the neonatal IS (16). It was shown that bacteria and immune factors can be transferred from mother to child (17, 18). In the absence of effective curative treatment, primary prevention strategies for FA represent a major opportunity.

Among allergy prevention strategies, prebiotic treatments are promising, as they can modulate the microbiota, epithelial barrier, and IS (19). Prebiotics are substrates selectively used by host microorganisms and that confer health benefits (20). Inulin and galacto-oligosaccharides (GOS) have been studied for their modulatory effects on the microbiota and have been shown to increase the relative abundances of beneficial microbial strains such as *Lactobacillus* and *Bifidobacterium* (21, 22). Prebiotics can also be fermented by specific commensal bacteria toward short-chain fatty acids (SCFAs), which influence many immune molecular and cellular processes and might help to prevent allergy development (23, 24).

We have recently published that prebiotic supplementation during pregnancy leads to the transmission of specific microbial and immune factors from mother to child, allowing the establishment of tolerogenic immune imprinting in the fetus that lasts over time (25). Indeed, this study highlighted the impact of GOS/inulin supplementation during gestation on 1) the modulation of the mother’s gut microbiota, leading to increased SCFA production; 2) the transfer of metabolites such as acetate and amino acids from mother to fetus in the amniotic fluid; 3) the tolerogenic environment induced *in utero* in feto-maternal tissues; and 4) the establishment of tolerogenic and Breg-mediated immune imprinting in the fetus that remains associated with Treg later in life. Moreover, Hogenkamps et al. and Fujiwara et al. demonstrated that antenatal prebiotic supplementation decreased airway hyper-reactivity and reduced sensitization in the allergic skin response using mouse model (26–28).

Herein, we hypothesized that the tolerogenic immune imprinting established by an antenatal prebiotic supplementation might prevent FA development. This preclinical study describes the effects of prebiotic (GOS/inulin) supplementation during gestation only on the occurrence of wheat FA in the pups and the associated immune and microbiota cellular and molecular mechanisms.

## Material and Methods

### Animal Studies

**Figure 1:**
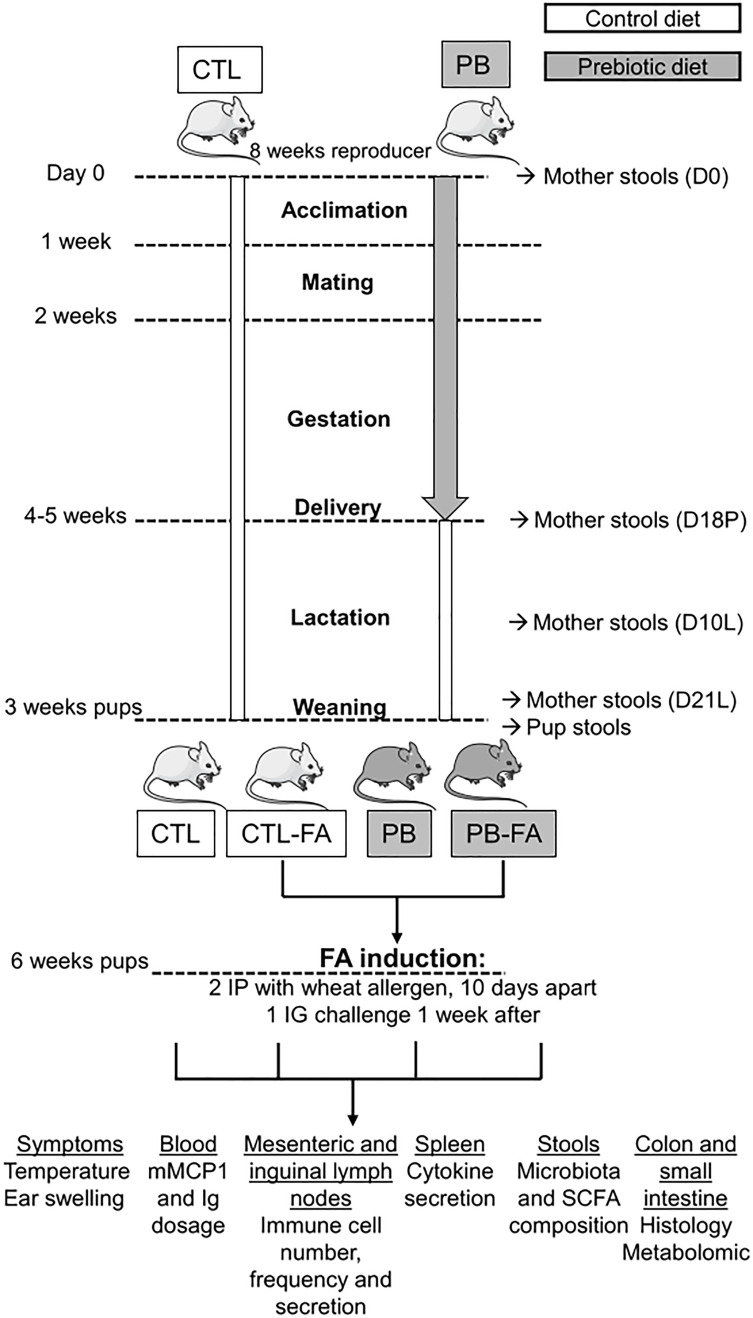
Experimental study design. Mothers were fed a diet supplemented with 4% galacto-oligosaccharide (GOS)/inulin prebiotics (PB) or a control (CTL) diet during acclimation, mating, and pregnancy (total of 4–5 weeks of supplementation). After delivery, mothers and pups were fed with a CTL diet. Wheat food allergy (FA) was induced in 3-week-old female pups born from mothers that received either a control diet (CTL-FA) and GOS/inulin diet (PB-FA). Pups were sensitized with two intraperitoneal (IP) injections 10 days apart with wheat allergen and aluminum hydroxide (Alum). One week after, pups were orally challenged by intragastric (IG) injection with wheat allergen. Pups born from mothers that received either a control diet or GOS/inulin diet, but were not sensitized to wheat, were used as controls (CTL and PB). Indicated samples were collected at different times during the protocol, and analysis was performed according to the sample.

Female and male BALB/cJRj mice were purchased at 8 weeks of age from Janvier Labs (France) and were used and housed in a ventilated cage system. The protocol was approved by the Ethics Committee on Animal Experimentation of the Pays de la Loire region (accreditation numbers: 14035 and 21419). Mice were fed either a control diet or a diet supplemented with 4% GOS (FrieslandCampina, Netherlands) and inulin (Orafti^®^ HP, Germany) in a 9:1 ratio (Safe, France) during acclimation (1 week), mating (1 week), and gestation (3 weeks) (**Figure 1**). After delivery, mothers and pups were fed a control diet. The food intake was similar between mothers on a control diet (31 ± 2 g/week) and mothers supplemented with prebiotic (31.8 ± 1 g/week). The weight gain of mothers and pups from the different groups was also similar. After weaning (at 21 days of life), the female offspring from each group of mothers (control or prebiotic) were randomized to abrogate the littermate effects. Wheat FA was induced in 3-week-old female pups born from mothers that received either a control diet (CTL-FA) or GOS/inulin diet (PB-FA). Pups born from mothers that received either a control diet or GOS/inulin diet, but were not sensitized to wheat, were used as controls (CTL and PB). For the mouse model of FA, 3-week-old female mice were intraperitoneally sensitized with two injections of 10 µg of wheat allergen^25^ dissolved in phosphate-buffered saline (PBS; Gibco, Thermo Fisher Scientific, France) v/v of aluminum hydroxide (Anhydrogel 2%, InvivoGen, USA) separated by 10 days. One week after the last injection, mice were orally challenged with 20 mg of wheat allergen by intragastric probe. We performed the full protocol twice, 4 months apart. The first protocol constituted seven CTL pups, 10 PB pups, six CTL-FA pups, and 10 PB-FA pups. The second protocol constituted three CTL pups, three PB pups, five CTL-FA pups, and three PB-FA pups.

### Food Allergy Symptoms and Euthanasia

To assess symptoms, rectal temperature and ear swelling were measured by rectal probes and micrometers, respectively, 30 min before and after challenge. Then, the mice were euthanized by intraperitoneal injection of Exagon^®^ (1 ml/1 kg, Axience, France).

### Histological Analysis of the Intestine

Jejunum sections were stored in 4% paraformaldehyde (Biovalley, France) before being embedded in paraffin and stained in a hematoxylin phloxine saffron solution. Histological scoring of intestinal integrity was established based on the following: 1) the degradation of intestinal villi (4 points), 2) the narrowing of intestinal crypt (4 points), and 3) the presence or absence of goblet cells (2 points). The total histological score represents the sum of all features evaluated (over 10 points) (29).

### Immunoglobulin Assay

Blood was collected after assessment of symptoms. Serum was obtained by incubating blood for 20 min on ice and then 20 min at 37°C and centrifuging for 15 min at 2,000 × *g*. The quantification of mouse-specific IgG1, IgG2a, IgA, and IgE was done using indirect ELISA as described by Adel-Patient et al. (30).

### mMCP-1 and Cytokine Assay

The concentration of mMCP-1 in serum samples was measured by ELISA Ready-SET-Go! (Fisher Scientific, USA) as described by the manufacturer. Mouse splenocytes were collected and cultured at 10^6^ cells/ml in Roswell Park Memorial Institute (RPMI) 1640 medium (Thermo Fisher, France) supplemented with 10% fetal calf serum (FCS), l-glutamine, and penicillin/streptomycin. They were stimulated by phorbol-12-myristate-13-acrylate (50 ng/ml, Sigma Aldrich, USA) and ionomycin (1 µg/ml, Sigma Aldrich) for 48 h. Thereafter, the concentrations of cytokines (IL-5, IL-10, IFN-γ, and TGF-β) in the culture medium were measured by a DuoSet ELISA kit (R&D Systems, USA) as described by the manufacturer.

### Analysis of the Fecal Microbiota Community by 16S rRNA Gene Survey Analysis

Total bacterial DNA was extracted from the collected stools according to the protocol described in Godon et al. (31). DNA concentration and integrity were determined spectrophotometrically using a NanoDrop instrument and visually by electrophoresis on a 1% agarose gel containing ethidium bromide. The DNA concentration values were between 0.6 and 1 µg/µl. The size distribution of the DNA extracted from the fecal samples estimated by agarose gel electrophoresis showed that most of the DNA was of high molecular weight (>20 kb) with no significant shearing. Taken together, these observations suggest that the extracted DNA was of good quality, suitable for downstream processing. The V3–V4 hyper-variable region of the 16S rRNA gene was amplified with the primers F343 (CTTTCCCTACACGACGCTCTTCCGATCTTACGGRAGGCAGCAG) and R784 (GGAGTTCAGACGTGTGCTCTTCCGATCTTACCAGGGTATCTAATCCT). The PCRs were performed using 10 ng of cecal DNA, 0.5 µM of primers, 0.2 mM of dNTP, and 0.5 U of the DNA-free Taq-polymerase, MolTaq 16S DNA Polymerase (Molzym). The amplifications were carried out using the following profile: 1 cycle at 94°C for 60 s, followed by 30 cycles at 94°C for 60 s, 65°C for 60 s, and 72°C for 60 s, and finishing with a step at 72°C for 10 min. The PCRs were sent to the GeT-PlaGe platform (INRA, France) for sequencing using Illumina MiSeq technology. Single multiplexing was performed using a homemade 6-bp index, which was added to R784 during a second PCR with 12 cycles using forward primer (AATGATACGGCGACCACCGAGATCTACACTCTTTCCCTACACGAC) and reverse primer (CAAGCAGAAGACGGCATACGAGAT-index-GTGACTGGAGTTCAGACGTGT). The resulting PCR products were purified and loaded onto the Illumina MiSeq cartridge according to the manufacturer’s instructions. The quality of the run was checked internally using PhiX, and then each paired-end sequence was assigned to its sample with the help of the previously integrated index. Each paired-end sequence was assembled using Flash software (32) using at least a 10-bp overlap between the forward and reverse sequences, allowing 10% of mismatch. The lack of contamination was checked with a negative control during the PCR (water as template). The quality of the stitching procedure was checked using four bacterial samples that are run routinely in the sequencing facility in parallel to the current samples.

### Short-Chain Fatty Acid Analysis of Fecal Samples

SCFA (acetate, propionate, and butyrate) content was determined by gas chromatography (Nelson 1020, Perkin-Elmer, France). The samples were extracted with water (wt g/vol), centrifuged at 17,000 × *g* for 10 min, and then the supernatant collected. The proteins were precipitated using a phosphotungstic acid-saturated solution. A volume of 0.1 ml of the supernatant was analyzed using a gas–liquid chromatograph (Autosystem XL; PerkinElmer, France). All samples were analyzed in duplicate. The data were collected, and peaks were integrated using Turbochromv6 software (PerkinElmer).

### Statistical Analyses of 16S rDNA Gene Sequences and Short-Chain Fatty Acids

Sequences were first analyzed using the FROGS pipeline to obtain the Operational Taxonomic Unit (OTU; or phylotypes) abundance table. The successive steps involved de-noising and clustering of the sequences into OTUs using SWARM; chimera removal using VSEARCh; taxonomic affiliation for each OTU using both RDPClassifier and National Center for Biotechnology Information (NCBI) Blast+ on Silva SSU 119 and 123. Statistical analyses were performed using “R” language and environment version 3.2.3. β-Diversity (UniFrac and weighted UniFrac dissimilarity) and α-diversity measurements and analysis of the differences in OTUs between samples were performed using the add-on package “Phyloseq.” Differences in the microbial communities between control and treated groups were evaluated using constrained analysis of principal coordinates and permutational multivariate ANOVA. Statistical differences between groups for individual OTU abundance and SCFA concentrations were calculated using the Mann–Whitney test with Benjamini–Hochberg false discovery rate (FDR) correction. Statistical significance was set at p < 0.05.

### NMR-Based Metabolic Fingerprints of Colon in Pups at 6 Weeks of Age

Colon samples (10–20 mg) were weight in a 2-ml tube for Fastprep homogenizer (Lysing Matrix D); 400 µl of methanol and 85 µl of water were added, and samples were homogenized two times using the FastPrep homogenizer for 30 s. Homogenates were transferred into glass tubes, 400 µl of dichloromethane and 200 µl of water were added, and samples were vortexed and centrifuged. Aqueous phases were collected and dried using the speedVac facility. Samples were reconstituted in 0.2 ml of phosphate buffer (0.2 M, pH 7.0), centrifuged (15 min, 5,500 × *g*, 4°C), and transferred into 3-mm NMR tubes. ^1^H NMR spectra were obtained at 300 K on a Bruker Avance III HD 600 MHz NMR spectrometer (Bruker BioSpin, Rheinstetten, Germany), operating at 600.13 MHz for the ^1^H resonance frequency using an inverse-detection 5-mm ^1^H–^13^C–^15^N–^31^P cryoprobe attached to a CryoPlatform. “Tuning” and “matching” of the probe, locking, shim tuning, pulsing (90°), and gain computations are automatically performed for each sample. ^1^H NMR spectra were acquired using the one-dimensional (1D) Carr–Purcell–Meiboom–Gill (CPMG) experiment with presaturation for water and macromolecule suppression (cpmgpr1d), with a spin-echo delay of 240 ms. A total of 128 transients were collected into 64k data points using a spectral width of 12 ppm, a relaxation delay of 5 s, and an acquisition time of 4.55 s. Prior to Fourier transform, an exponential line broadening function of 0.3 Hz was applied to the free induction decay (FID). All NMR spectra were phase- and baseline-corrected and referenced to the chemical shift of TSP (0 ppm) using Topspin (V3.2, Bruker BioSpin, Germany). The NMR spectra were then divided into fixed-size buckets (0.01 ppm) between 9 and 0.5 ppm using AMIX software (v3.9.15, Bruker), and the area under the curve was calculated for each bucket. The regions including residual water (5.2–4.4 ppm) were removed. Integrations were normalized according to the total intensity. A principal component analysis (PCA) of metabolic fingerprints followed by a partial least squares discriminant analysis (PLS-DA) was then performed to evaluate discrimination between supplementation levels. These multivariate methods were described by Cabaton (33). NMR buckets with variable importance in projection (VIP) >1.0 were selected as discriminants. Finally, a non-parametric univariate Wilcoxon test was performed on metabolic features from multivariate analysis. The FDR was applied to take into account multiple testing and avoid false positives. Statistical analyses were conducted with Simca software (V15; Umetrics AB, Umea, Sweden) and R (in house scripts and the ropls package (34).

### Flow Cytometry

Mouse spleen and lymph nodes (mesenteric for the T-cell populations and inguinal for B-cell populations) were harvested and crushed to obtain a single-cell suspension. In the spleen, red blood cells were removed by a red blood lysis buffer (Invitrogen, Life Technologies, USA). A total of 1 × 10^6^ cells were transferred to 96-well plates and stimulated for 5 h with RPMI, 5% FCS, 1% penicillin/streptavidin, phorbol-12-myristate-13-acrylate (50 ng/ml), and ionomycin (1 µg/ml) at 37°C, 5% CO_2_. Brefeldin A and monensin (1 mg/ml, BD Biosciences, USA) were also added 2 h after stimulation. Cells were labeled with surface markers (CD3-FITC, CD4-PerCP-Cy5.5, CD8a-PE-Cy7, CD25-Brilliant Vio510; CD19-PerCP vio770, CD9 PE-vio770, BD Biosciences and Miltenyi Biotec, France) with the Fc blocking antibody anti-CD16/CD32 (BD Biosciences). Cells were fixed and permeabilized with a Fixation/Permeabilization Solution Kit (BD Biosciences) to perform intracellular labeling (Fox-P3-PE, IL-4-APC, IFN-γ-APC-Cy7, BD Biosciences). Cells were analyzed by flow cytometry on a BD FACSCanto™ II Cell Analyzer (BD Biosciences). Data were acquired with Diva 8.0 software and analyzed with FlowJo Software v10 (TreeStar, Williamson Way, USA).

### Statistical Analysis

Statistical analyses were performed using GraphPad Prism software, version 5.03 (La Jolla, CA, USA). Values were compared using the Wilcoxon or Mann–Whitney test. A two-sided p < 0.05 was considered statistically significant (*p < 0.05; **p < 0.01; ***p < 0.001; ****p < 0.0001). Results are expressed as the mean ± standard error.

## Results

### The Composition and Function of the Mother’s Gut Microbiota Are Modified During Galacto-Oligosaccharide/Inulin Supplementation But Progressively Converge to Control Levels Once Supplementation Is Stopped

**Figure 2:**
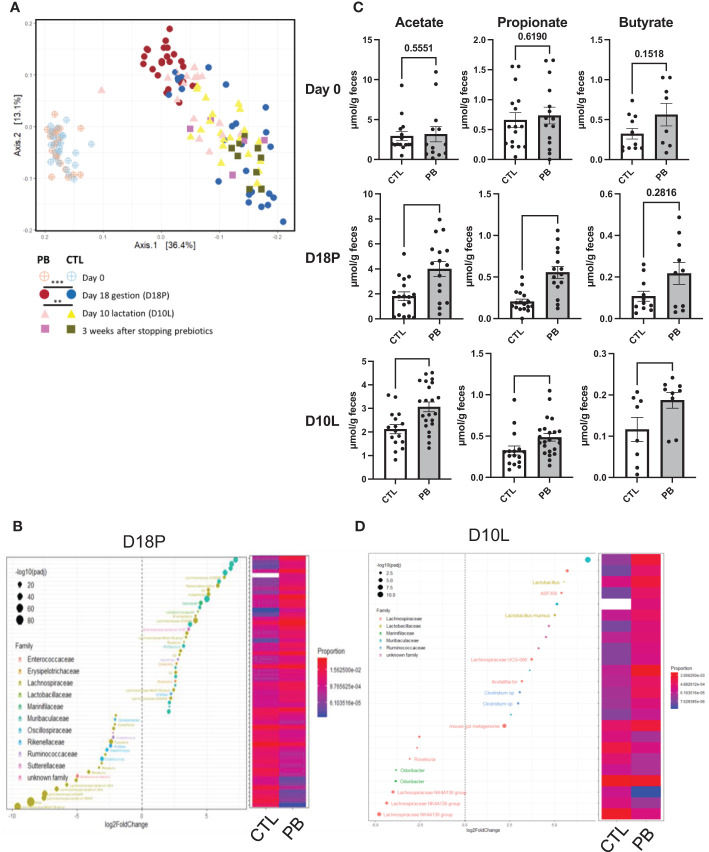
A prebiotic diet modifies the fecal microbiota composition and short-chain fatty acid (SCFA) levels during and after supplementation. **(A)** Analysis of the β-diversity by unweighted UniFrac-based principal coordinates analysis (PCoA) from stools of dams fed a control (CTL) or prebiotic (PB) diet (n = 20 per group). [**(B, D)**, left figure] Graphic representation of differentially abundant Operational Taxonomic Units (OTUs) (FC > I2I; adjusted posttest p-value <0.05) between CTL and PB mothers **(B)** at 18 days of gestation (D18P) and **(D)** at 10 days of lactation (D10L). The OTUs shown are present in 75% of samples and present at least 1% in one of the CTL or PB groups. Each OTU is colored according to its taxonomic classification at the family level. Taxonomy at the genus or species level is also indicated, when available, next to each OTU. Log2FoldChange (FC) is plotted on the x-axis. The diameter of the circle drawn for each OTU is related to the value of the adjusted posttest p-value. [**(B, D)**, right Figure] Heatmap representing the relative abundances of differentially abundant OTUs in the stools of dams fed the CTL or PB diet. **(C)** Evaluation of acetate, propionate, and butyrate levels in mother’s stools at day 0, D18P, and D10L. SCFA data are displayed as the mean min-to-max values (n = 8 to 20 animals per group). SCFA p-values were determined using the Mann–Whitney test (*p < 0.05, **p < 0.01, ***p < 0.001).

To evaluate the impact of GOS/inulin supplementation on the microbiota of pregnant mice, stools were collected on day 0 before GOS/inulin supplementation (D0) and on day 18 corresponding to the end of gestation (D18P). Stools were analyzed by 16S rRNA sequencing to evaluate microbial α- and β-diversity. No change in α-diversity (microbial richness) was seen between the CTL (control) and PB (prebiotic) groups at all time points as previously described (35) (**Supplementary Figures 1A–E**). β-Diversity of the fecal microbiota was also not altered between the CTL and PB groups on D0 but was significantly different between the CTL and PB groups on D18P (**Figure 2A**) (p < 0.001). A total of 128 OTUs were significantly increased in the microbiota of the PB-supplemented mice (listed in **Supplementary Table 1**). Specifically, the relative abundance of the phyla Bacteroidetes, Actinobacteria, and Proteobacteria was increased, whereas the abundance of Desulfobacteria and Firmicutes was decreased in fecal microbiota of PB-supplemented mice (**Table 1**). Among these phyla, 15 families were modulated, with an increase in Muribaculaceae, Atopobiaceae, Lactobacillaceae, and Erysipelotrichaceae and a decrease in Bifidobacteriaceae, Rikenellaceae, and Ruminococcaceae (**Table 1**). More specifically, OTUs from the Muribaculaceae and Lactobacillaceae families were the most increased in PB-supplemented mothers, and a strong reshaping of Lachnospiraceae OTUs was observed between the two groups (**Figure 2B** and **Supplementary Table 1**). To evaluate the functional impact of these changes in fecal microbiota, SCFA (acetate, propionate, and butyrate) concentrations were measured by gas chromatography. As expected, the SCFA levels were similar between the two groups on D0 (**Figure 2C**). However, at D18P, the modification of the gut microbiota in the PB-supplemented group was associated with a significant increase in the acetate and propionate levels. The butyrate level was not affected by prebiotic supplementation at D18P (acetate 1.8 ± 0.3 *vs*. 4.0 ± 0.6 µmol/g feces, p = 0.006; propionate 0.2 ± 0.03 *vs*. 0.6 ± 0.1 µmol/g feces, p = 0.0001; for CTL and PB, respectively).

**Table 1:** Summary table of relative abundance of phyla and families of fecal microbiota based on 16S rRNA sequencing (region V3–V4).

	Dams								Pups			
	Day 0		D18P		D10L		End of lactation		NON-FA		FA	
Actinobacteria	0.4		*	+	0.7		0.1		*	+	*	+
*Atopobiaceae*	0.5		**	+	0.8		0.3		0.2		0.07	
*Bifidobacteriaceae*	ND		**	-	0.2		ND		*	+	ND	
*Eggerthellaceae*	0.4		1.0		0.6		0.6		0.3		0.2	
Bacteroidetes	0.1		**	+	0.4		0.9		*	-	0.4	
*Bacteroidaceae*	0.1		*	-	1.0		0.4		0.5		0.3	
*Marinifilaceae*	0.9		0.6		0.2		0.6		0.2		0.7	
*Muribaculaceae*	0.1		***	+	0.9		**	-	0.2		0.4	
*Prevotellaceae*	0.3		0.5		0.6		0.3		0.7		*	+
*Rikenellaceae*	0.1		**	-	0.5		0.3		0.1		0.07	
*Tannerellaceae*	*	**-**	*	-	*	+	0.4		0.2		0.8	
Desulfobacteria	0.3		***	-	*	-	1.0		*	+	1.0	
*Desulfovibrionaceae*	0.2		***	-	0.05		1.0		*	+	1.0	
Firmicutes	0.2		*	-	0.07		0.9		0.2		0.4	
*Eubacterium*	0.9		0.1		0.1		0.4		ND		ND	
*Butyricicoccaceae*	0.6		**	-	0.7		0.4		0.2		0.1	
*Enterococcaceae*	0.2		***	-	0.8		0.7		0.9		0.1	
*Erysipelotrichaceae*	0.6		***	+	0.1		0.6		ND		ND	
*Lachnospiraceae*	0.2		0.3		0.6		0.8		0.09		0.4	
*Lactobacillaceae*	0.8		***	+	**	+	0.2		ND		**	+
*Oscillospiraceae*	0.2		***	-	0.5		0.6		1.0		0.4	
*others*	0.6		0.2		0.3		0.9		0.2		0.4	
*Peptostreptococcaceae*	ND		0.06		0.5		0.9		0.2		*	-
*Ruminococcaceae*	0.7		**	-	1.0		0.7		0.2		0.8	
*Unknown*	0.8		*	+	**	+	0.5		0.8		0.06	
Proteobactéria	0.05		***	+	*	+	0.2		*	+	0.2	
*Enterobacteriaceae*	0.9		0.7		0.8		ND		0.9		0.2	
*Sutterellaceae*	0.1		***	+	**	+	0.1		*	+	0.1	
*Unknown*	0.6		0.5		0.07		0.4		ND		ND	

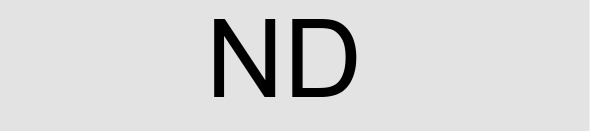
not determined.

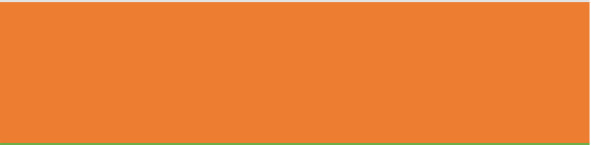
Differential microbial abundance found in dams and offspring according to gestation diet.

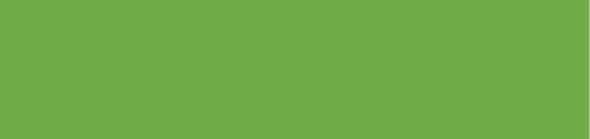
Differential microbial abundance found in dams according to gestation diet.

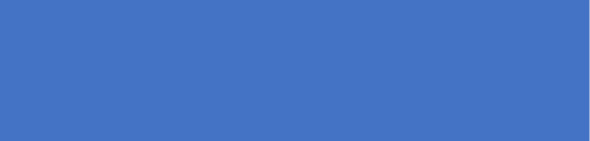
Differential microbial abundance found in offspring according to gestation diet.

“+” means increased and “−” means decreased in the prebiotic supplemented group compared with the control group. p-Values were determined using the Mann–Whitney test.

*p < 0.05.

**p < 0.01.

***p < 0.001.

To determine if these changes in microbiota diversity lasted when supplementation was stopped, stool samples were collected on day 10 of lactation (mid-lactation D10L, 10 days after stopping prebiotics) and at the end of lactation (3 weeks after stopping the prebiotics, D21L). At D10L, the intestinal β-diversity of PB-supplemented mice remained significantly different from that of the CTL group but tended to converge toward the gut microbiota of control mice (p < 0.002) (**Figure 2A**). At this time, only 16 different OTUs were overabundant in the stool of PB-supplemented mice, from the Muribaculaceae, Lactobacillaceae, and Ruminococcaceae families (**Figure 2D** and **Supplementary Table 2**). At the family level, Tannerelaceae, Lactobacillaceae, and Sutterellaceae were overrepresented in PB mother’s stools (**Table 1**). The Proteobacteria phylum was always increased. Despite stopping prebiotic supplementation, acetate, propionate, and butyrate levels were increased in the PB-supplemented mothers (acetate 2.12 ± 0.2 *vs*. 3.22 ± 0.3 µmol/g feces, p = 0.006; propionate 0.32 ± 0.05 *vs*. 0.48 ± 0.04 µmol/g feces, p = 0.007; butyrate 0.11 ± 0.02 *vs*. 0.18 ± 0.01 µmol/g feces, p = 0.020, for CTL and PB, respectively) (**Figure 2C**). Finally, at the end of lactation, the fecal microbiota from both groups were similar, except for three OTUs from the Lachnospiraceae and Rikenellaceae families, which were still higher in the PB group than in the CTL group (**Figure 2A**, **Table 1**, **Supplementary Figure 1F**, **Supplementary Table 3**). Unfortunately, SCFA could not be quantified for this time point. In summary, the supplementation of the maternal diet with prebiotics enriches the fecal microbiota of dams in particular Bacteroidetes, Actinobacteria, and Proteobacteria and, more specifically, with families such as Muribaculaceae and Lactobacillaceae. These effects weakly persisted at least 10 days after stopping GOS/inulin consumption.

### The Gut Microbiota of 6-Week-Old Pups Born From Galacto-Oligosaccharide/Inulin-Supplemented Mothers Differs From That of Pups Born From Mothers Fed a Control Diet

**Figure 3:**
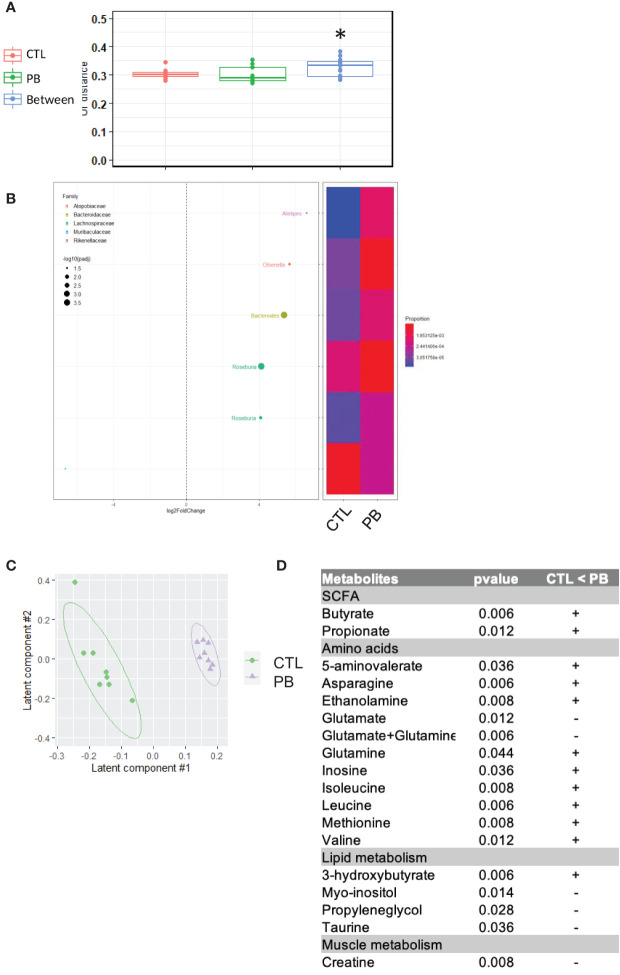
Offspring from prebiotics- and control-supplemented dams have different microbiota at 6 weeks of age. **(A)** β-Diversity as measured by UniFrac distances from control (CTL) or prebiotic (PB) pups based on 16S rRNA gene sequencing at 6 weeks of age. Within- and between-group dissimilarities were tested by permutational multivariate ANOVA (PERMANOVA) (*p < 0.05; n = 20 per group). (**B**, left figure) Graphic representation of differentially abundant Operational Taxonomic Units (OTUs) (FC > I2I; adjusted posttest p-value <0.05) between CTL and PB pups at 6 weeks of age. Each OTU is colored according to its taxonomic classification at the family level. Taxonomy at the genus or species level is also indicated, when available, next to each OTU. Log2FoldChange (FC) is plotted on the x-axis. The diameter of the circle drawn for each OTU is related to the value of the adjusted posttest p-value. (**B**, right figure) Heatmap representing the relative abundance of differentially abundant OTUs in pups at 6 weeks of age in stools of dams fed the CTL or PB diet. **(C)** Two-dimensional partial least squares discriminant analysis (PLS-DA) score plot of OSC-filtered and Pareto-scaled ^1^H NMR integrated spectra of colon samples (green circle: control, n = 8; purple triangle: supplementation, n = 8). **(D)** Table of discriminant metabolites measured in colon between CTL and PB pups.

Our data support a strong modification of maternal fecal microbiota composition and SCFA levels during pregnancy and mid-lactation. To address the possibility of specific microbial transmission from mothers to offspring, we investigated the fecal microbiota composition by 16S rRNA sequencing. We observed a significant difference in the β-diversity of the fecal microbiota between offspring of PB-supplemented and CTL mothers (**Figure 3A**). Similar to that of their mothers at D18P, the microbiota of PB pups showed an increased relative abundance of Actinobacteria and Proteobacteria (**Table 1**). Specifically, Bifidobacteriaceae, Desulfovibrionaceae, and Sutterellaceae families were higher in PB pups than in CTL pups. The relative abundance of five identified OTUs from Rikenellaceae, Atopobiaceae, Bacteroidaceae, and Lachnospiraceae families were higher in the PB group than in the CTL group (**Figure 3B** and **Supplementary Table 4**). Again, to evaluate the functional impact of these changes in fecal microbiota, we determined the concentration of several metabolites by NMR in colon tissue of CTL and PB pups at 6 weeks of age. In the colon, PLS-DA showed a clear separation between the CTL and PB pups samples (**Figure 3C**). A total of 164 features were selected based on the VIP index (VIP > 1) together with a Wilcoxon non-parametric test. In line with data from the mothers, the levels of the SCFA butyrate and propionate were significantly higher in the colons of PB pups than in CTL pups (**Figure 3D**). Eighteen other metabolites were also found in higher concentration in the colon of PB pups compared with CTL pups. These metabolites belong to different categories, such as amino acid metabolism, the citric acid cycle, lipid metabolism, and muscle metabolism. These results demonstrate that an antenatal prebiotic diet influences the fecal microbial composition of pups at least until 6 weeks of age, as well as different metabolites, including SCFA.

### Galacto-Oligosaccharide/Inulin Supplementation During Pregnancy Induces a Tolerogenic Immune Environment in Pups

**Figure 4:**
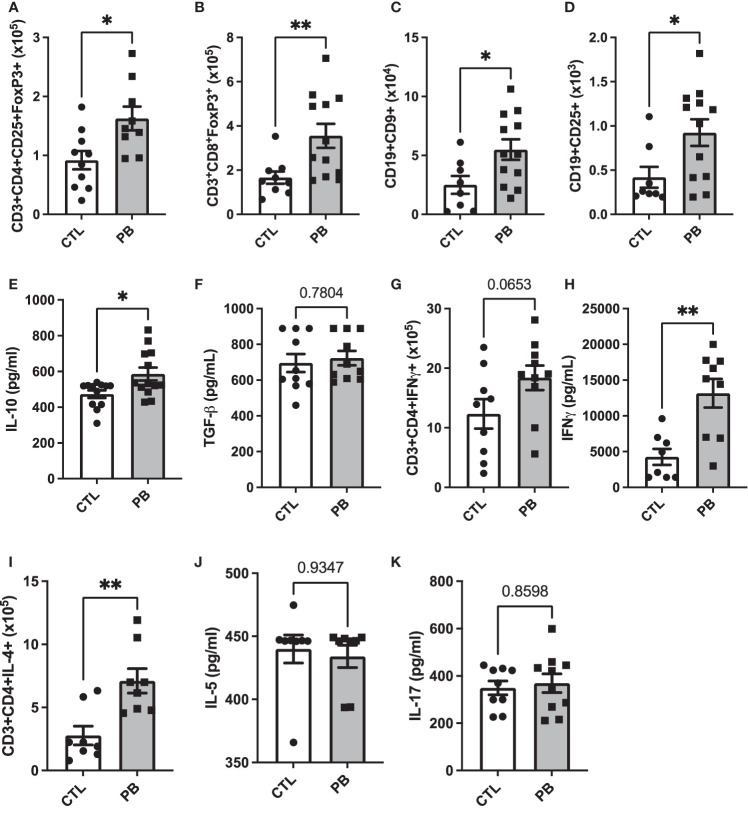
Prebiotics supplementation during pregnancy sets up a pro-tolerogenic environment. Total number of **(A)** CD3^+^CD4^+^CD25^+^FoxP3^+^, **(B)** CD3^+^CD8^+^FoxP3^+^, **(C)** CD19^+^CD9^+^, **(D)** CD19^+^CD25^+^, **(G)** CD3^+^CD4^+^IFN-γ^+^, and **(I)** CD3^+^CD4^+^IL-4^+^ and cells in mesenteric (for T cells) and inguinal (for B cells) lymph nodes. Splenocytes secretion of **(E)** IL-10, **(F)** TGF-β, **(H)** IFNγ, **(J)** IL-5, and **(K)** IL-17. All data are displayed as the mean ± standard error (n = 8 to 12 animals per group); p-values were determined using the Mann–Whitney test (*p < 0.05, **p < 0.01).

To understand the impact of prebiotic supplementation during pregnancy on the offspring’s IS, we analyzed the number of several immune cell populations from mesenteric (T cells) and inguinal (B cells) lymph nodes: CD3^+^CD4^+^CD25^+^Foxp3^+^ Treg, CD3^+^CD8^+^Foxp3^+^ Treg, CD9^+^ Breg, CD25^+^ Breg, CD3^+^CD4^+^IFN-γ^+^ Th-1 cells, and CD3^+^CD4^+^IL-4^+^ Th-2 cells by flow cytometry (gating strategy **Supplementary Figures 2A, B** for T- and B-cell subsets, respectively). We also quantified the concentration of IL-5 (Th2 cytokine), IFN-γ (Th1 cytokine), IL-17 (Th17 cytokine), IL-10, and TGF-β (regulatory cytokines) in spleen supernatants by ELISA. PB pups showed significantly higher numbers of CD3^+^CD4^+^CD25^+^Foxp3^+^ and CD3^+^CD8^+^Foxp3^+^ Treg than CTL pups (0.9 × 10^5^ ± 0.2 vs. 1.4 × 10^5^ ± 0.2 cells for CTL and PB CD3^+^CD4^+^CD25^+^Foxp3^+^ Treg, p = 0.01) (**Figure 4A**) (1.7 × 10^5^ ± 0.3 *vs*. 3.5 × 10^5^ ± 0.5 cells for CTL and PB CD3^+^CD8^+^Foxp3^+^ Treg, p = 0.007) (**Figure 4B**). However, the frequency of these regulatory cells was the same in both groups (**Supplementary Figures 3A, B**). The CD9^+^ Breg number increased in the PB group (3.5 × 10^4^ ± 0.9 *vs*. 5.9 × 10^4^ ± 0.9 cells for CTL and PB, respectively; p = 0.04) (**Figure 4C**) such as the frequency (2.2% ± 1.6 *vs*. 4.1% ± 1.3 for CTL and PB, respectively; p = 0.007) (**Supplementary Figure 3C**). CD25^+^ Breg number and frequency also increased in the PB group (3.2 × 10^3^ ± 0.8 *vs*. 10.1 × 10^3^ ± 2.1 cells for CTL and PB, respectively; p = 0.03) (**Figure 4D**). The increased number and frequency of regulatory immune cell subsets in PB pups were confirmed by the increased secretion of IL-10 in the PB group compared with the CTL group in spleen supernatant (473 ± 21.1 *vs*. 585.4 ± 35.9 pg/ml for CTL and PB, respectively; p = 0.03) (**Figure 4E**). The levels of TGF-β secretion were similar in both groups (**Figure 4F**). Concerning the Th cell balance, the CD3^+^CD4^+^IFN-γ^+^ Th-1 cell number only tended to increase in the PB group (**Figure 4G**), but the secretion of IFN-γ was significantly higher (3,810 ± 1,080.5 *vs*. 11,870.6 ± 2,324.2 pg/ml for CTL and PB, respectively; p = 0.004) (**Figure 4H**). The frequency was similar in both groups (**Supplementary Figure 3E**). The CD3^+^CD4^+^IL-4^+^ Th-2 cell number and frequency in the PB group were significantly increased compared with those in the CTL group (2.78 × 10^5^ ± 0.7 *vs*. 6.78 × 10^5^ ± 0.9 in CTL *vs*. PB, respectively, p = 0.007) (**Figure 4I**, **Supplementary Figure 3F**), but the level of IL-5 secretion was similar between both groups (**Figure 4J**). Concerning the IL-17 secretion, no difference was observed between both groups (**Figure 4K**). Taken together, these results demonstrate the establishment of tolerogenic immune environment in pups birthed from PB-supplemented mothers.

### Maternal Intake of Galacto-Oligosaccharide/Inulin Protects Offspring From Wheat-Food Allergy Symptoms

**Figure 5:**
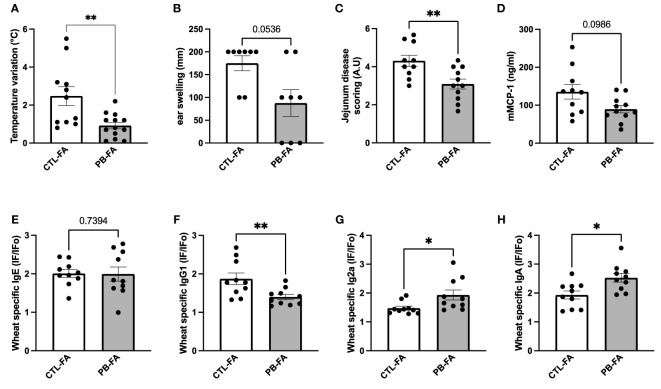
Offspring from galacto-oligosaccharide (GOS)/inulin-supplemented mothers are protected from wheat food allergy (FA). **(A)** Change in body temperature and **(B)** ear thickness variation were measured 30 min before and 30 min after challenge. **(C)** Jejunum disease scoring was determined by histological section. Serum levels of **(D)** mMCP-1, **(E)** wheat-specific IgE, **(F)** IgG1, **(G)** IgG2a, and **(H)** IgA. The results are expressed as the ratio of fluorescence intensities measured with wheat (IF) to fluorescence intensity measured with PBS (IFo). All data are displayed as the mean ± SEM (n = 11 to 13 animals per group). p-values were determined using the Mann–Whitney test (*p < 0.05; **p < 0.01).

Considering the observed effects of PB on the IS and the microbiota of the offspring, we wanted to determine whether prebiotic supplementation can prevent FA development. To this end, pups were sensitized to wheat by two intraperitoneal injections and were challenged with wheat by gavage 1 week after. Symptoms (rectal temperature, ear thickness, and jejunal barrier degradation) and allergic biomarkers (wheat-specific Ig and mMCP-1) were evaluated after oral provocation with wheat allergen. Interestingly, a mother’s exposure to GOS/inulin during gestation prevented the severity of FA symptoms. PB-FA offspring demonstrated a significant decrease in rectal temperature variation (2.5°C ± 0.5°C vs. 0.9°C ± 0.2°C for CTL-FA and PB-FA, respectively, p = 0.009) (**Figure 5A**) and a tendency to ear thickness swelling reduction compared with CTL-FA offspring after allergen challenge (175 ± 16.4 vs. 87.5 ± 29.5 mm for CTL-FA and PB-FA, respectively, p = 0.053) (**Figure 5B**). An improvement in the deterioration of the jejunal barrier was observed in pups exposed *in utero* to prebiotics (score of 4.3 ± 0.3 *vs*. 3.1 ± 0.3 for CTL-FA and PB-FA, respectively, p = 0.009) (**Figure 5C**). The mMCP-1 concentration in the sera in the PB-FA pups tended to be reduced compared with that in the CTL-FA pups (**Figure 5D**). The wheat-specific IgE levels among the two FA groups were similar (**Figure 5E**), but the wheat-specific IgG1 levels were significantly decreased in PB-FA compared with CTL-FA (1.9 ± 0.1 *vs*. 1.4 ± 0.1 IF/IFo, for CTL-FA and PB-FA, respectively, p = 0.009) (**Figure 5F**). Remarkably, wheat-specific IgG2a and wheat-specific IgA in PB-FA were significantly increased compared with those in CTL-FA (IgG2a: 1.5 ± 0.1 vs. 1.9 ± 0.2 IF/IFo, p = 0.01) (**Figure 5G**) (IgA: 1.9 ± 0.1 *vs*. 2.5 ± 0.1 IF/IFo, p = 0.01, in CTL-FA and PB-FA, respectively) (**Figure 5H**). Together, these results demonstrate a protective effect of antenatal GOS/inulin supplementation against FA symptoms in offspring that is associated with increased levels of anti-inflammatory Ig, such as IgG2a and IgA and decreased levels of the pro-allergic IgG1.

### Food Allergy Pups Present a Different Gut Microbiota Composition Depending on Maternal Diet

**Figure 6:**
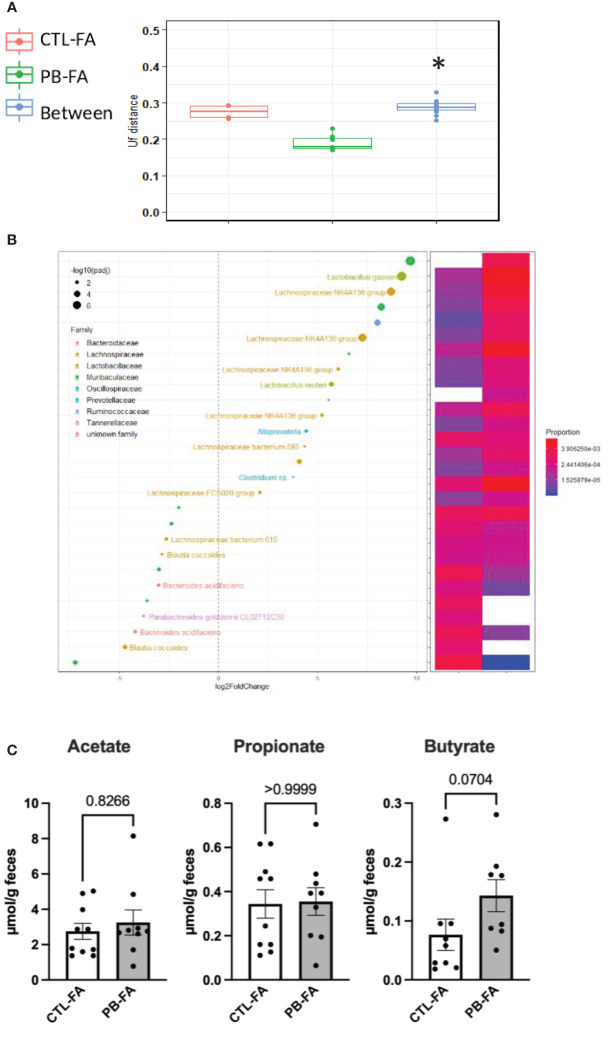
Galacto-oligosaccharide (GOS)/inulin supplementation during pregnancy modifies the fecal microbiota and the levels of short-chain fatty acids (SCFAs) in food allergy (FA) offspring. **(A)** β-Diversity as measured by UniFrac distances from control (CTL-FA) and prebiotic (PB-FA) allergic pups based on 16S rRNA gene sequencing at 6 weeks of age. Within- and between-group dissimilarities were tested by permutational multivariate ANOVA (PERMANOVA) (n = 9–10 pups per group). (**B**, left figure) Graphic representation of differentially abundant Operational Taxonomic Units (OTUs) (FC > I2I; adjusted posttest p-value < 0.05) in 6-week-old allergic pup microbiota. Each OTU is colored according to its taxonomic classification at the family level. Taxonomy at the genus or species level is also indicated, when available, next to each OTU. Log2FoldChange (FC) is plotted on the x-axis. The diameter of the circle drawn for each OTU is related to the value of the adjusted posttest p-value as specified in the figure. (**B**, right figure) Heatmap representing the relative abundances of differentially abundant OTUs in 6-week-old allergic pup microbiota. **(C)** Fecal level of acetate, propionate, and butyrate of allergic pups at 6 weeks of age. Data are displayed as the mean min-to-max values (n = 8 to 10 animals per group); p-values were determined using the Mann–Whitney test (*p < 0.05).

To determine the mechanism for the protective effects of antenatal PB supplementation on FA, stools from pups were collected at the end of the FA protocol (6 weeks of age), and the fecal microbiota composition and SCFA concentration were investigated as described previously. FA pups born from GOS/inulin-supplemented mothers had a different gut microbiota at 6 weeks of age. Indeed, the gut microbiota β-diversity of the CTL-FA and PB-FA groups was significantly different, as shown in **Figure 6A** (p = 0.01). The relative abundance of the Actinobacteria phylum and Lactobacillaceae and Prevotellaceae families in the PB-FA group was significantly increased compared with that in the CTL-FA group (**Table 1**). Similarly, we identified an increased abundance of specific OTUs from Ruminococcaceae, Lactobacillaceae, and Muribaculaceae families in the PB-FA group compared with the CTL-FA group (**Figure 6B** and **Supplementary Table 5**). With respect to SCFA levels, no difference was observed between the two groups (**Figure 6C**). Overall, these results suggest that maternal supplementation with prebiotics modulates the fecal microbiota composition in FA offspring.

### Antenatal Exposure to Galacto-Oligosaccharide/Inulin Tends to Reduce the Th2 Response and Enhances Tolerance in Food Allergy Offspring

**Figure 7:**
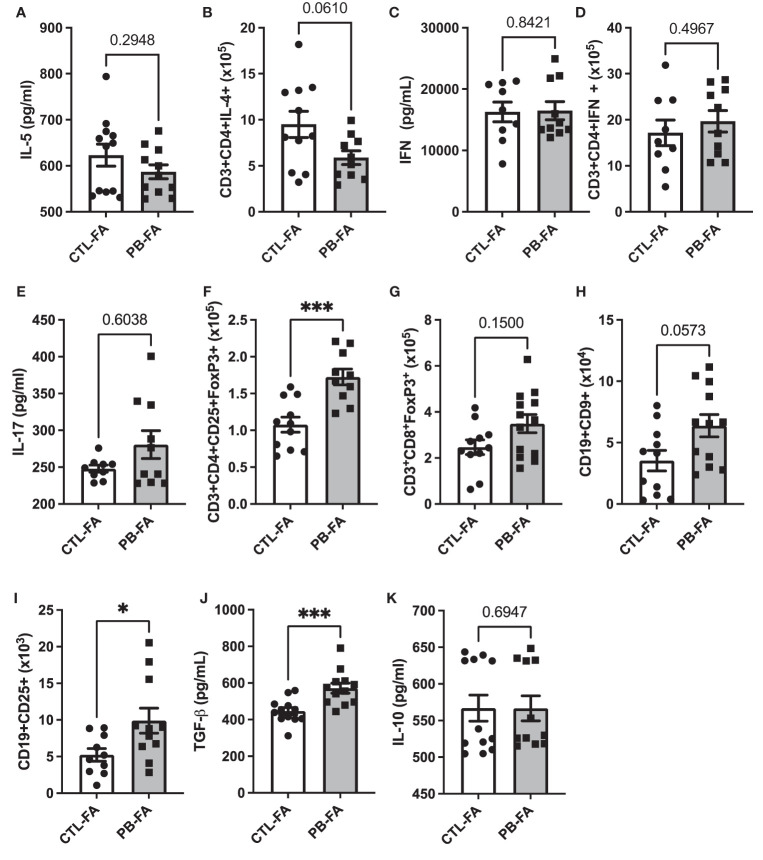
In food allergy (FA) offspring, helper T-cell responses and immune tolerance markers are modulated antenatal exposure to prebiotics. Splenic cytokine secretion of **(A)** IL-5, **(C)** IFN-γ, **(E)** IL-17, **(J)** TGF-β, and **(K)** IL-10 and total count of **(B)** CD3^+^CD4^+^IL-4^+^, **(D)** CD3^+^CD4^+^IFN-γ^+^, **(F)** CD3^+^CD4^+^CD25^+^FoxP3^+^, **(G)** CD3^+^CD8^+^FoxP3^+^, **(H)** CD19^+^CD9^+^, and **(I)** CD19^+^CD25^+^ cells in mesenteric (for T cells) and inguinal (for B cells) lymph nodes after wheat challenge. All data are displayed as the mean ± SEM (n = 9–12 animals per group); p-values were determined using the Mann–Whitney test (*p < 0.05, ***p < 0.001).

To understand the protective effect of an antenatal GOS/inulin diet on FA prevention, we evaluated the presence of different immune cell populations and the expression of their associated biomarkers (gating strategy **Supplementary Figures 2A, B** for T and B cells subsets, respectively). We analyzed the number of Th and regulatory cells in lymph nodes by flow cytometry, and we measured the concentrations of several cytokines by ELISA: IL-5, IFN-γ, IL-17, IL-10, and TGF-β in the culture medium of stimulated splenocytes. IL-5 secretion from splenocytes was similar between the CTL-FA and PB-FA groups (**Figure 7A**), but the number of CD3^+^CD4^+^IL-4^+^ Th2 cells in the PB-FA group tended to decrease compared with the CTL-FA group (**Figure 7B**). IFN-γ secretion and the number of CD3^+^CD4^+^IFN-γ^+^ Th-1 cells were not significantly different between the CTL-FA and the PB-FA groups (**Figures 7C, D**), nor was IL-17 secretion (**Figure 7E**). The number of CD3^+^CD4^+^CD25^+^FoxP3^+^ Treg was significantly increased in the PB-FA group compared with the CTL-FA group (1.1 × 10^5^ ± 0.2 vs. 1.7 × 10^5^ ± 0.2, CTL-FA and PB-FA, respectively, p = 0.0006) (**Figure 7F**) such as the frequency (1.3% ± 0.3 cells *vs*. 1.6% ± 0.3 for CTL and PB, respectively; p = 0.03) (**Supplementary Figure 4A**). CD3^+^CD8^+^FoxP3^+^ Treg tended to be increased in PB-FA pups compared with the CTL-FA pups (**Figure 7G**). CD9^+^ Breg number tended to be increased in the PB-FA pups compared with the CTL-FA pups (**Figure 7H**), but the frequency was significantly higher (2.2% ± 1.5 cells vs. 4% ± 1.7 for CTL and PB, respectively; p = 0.04) (**Supplementary Figure 4C**). CD25^+^ Breg number was significantly increased in the PB-FA groups (5.8 × 10^5^ ± 1.0 *vs*. 9.9 × 10^5^ ± 1.6 CTL-FA and PB-FA, respectively, p = 0.03) (**Figure 7I**). The enhancement of the regulatory immune response in pups exposed to antenatal GOS/inulin supplementation was confirmed by the increased secretion of TGF-β in the PB-FA group compared with the CTL-FA group (456.8 ± 18.2 *vs*. 572.9 ± 27.4 pg/ml for CTL-FA and PB-FA, respectively, p = 0.0007) (**Figure 7J**). However, no difference in IL-10 secretion was observed (**Figure 7K**). Overall, these results highlight that antenatal supplementation with prebiotics tends to decrease the Th2 response, induces an immune tolerance response in offspring, and thereby protects them from FA development.

## Discussion

In accordance with the DOHaD concept, food is a key environmental factor with the capacity to modulate mother–child interactions during the first 1,000 days of life, a period crucial for the proper establishment of the IS, the microbiota, and the future health of the child (14, 15). We demonstrate that GOS/inulin supplementation during gestation reshapes the maternal fecal microbiota and thereby modifies the fecal microbiota in the offspring. These changes promote immune tolerance and mitigate the development of FA in offspring.

Regarding the gut microbiota, our study shows that a GOS/inulin diet during gestation induces a strong modulation of β-diversity. Our data are in line with studies showing that a GOS/inulin intervention increases the relative abundance of Actinobacteria and Bacteroidetes phyla and Lactobacillaceae and Muribaculaceae families (22, 36–38). This microbial profile was previously associated with the absence of FA in infants (39). We did not observe an increase in Bifidobacteriaceae abundance as described in humans after PB consumption (21, 22), which may be explained by the very low abundance of this family in the adult murine microbiota (40). After prebiotic supplementation was stopped, the mother’s gut microbiota was quite resilient, as previously described (35). Our results also highlight an increase of gut microbiota functionality with an increased concentration of acetate and propionate in the stools. Indeed, it is known that Bacteroidetes, Actinobacteria, Lactobacillaceae, and Muribaculaceae are strong producers of SCFAs (41, 42). A transfer of bacteria, bacterial DNA, or bacterial metabolites between mother and child is suspected *in utero* (43–45) and was demonstrated during delivery (46).

In accordance with data from the mothers, the relative abundance of Actinobacteria and Proteobacteria was significantly increased in pups from PB-supplemented mothers at 6 weeks of age. Unlike their mothers, pups from PB-supplemented mothers also showed an increased abundance of the Bifidobacteriaceae family. Indeed, prebiotic supplementation induces a specific signature that is characterized by an increase in Bifidobacteriaceae (47). These results suggest the transmission of a microbial imprint from mother to child during delivery and/or gestation and the persistence of this imprint until at least 6 weeks of life. The modification of the fecal microbiota composition was also associated with differences in the relative abundance of various metabolites such as SCFAs, amino acids, or lipids in the colon. Thus, prebiotic supplementation during pregnancy not only modulates the composition of the microbiota but also modulates the functionality of the microbiota as characterized by the secretion of different metabolites.

Microbial transfers between mother and child might trigger immune responses in the fetus and the newborn that might shape the infant’s IS development, the notion of “early life imprinting” of the IS (48). In this context, we already published that prebiotic supplementation during gestation increased the frequency of tolerogenic immune cells (Treg and Breg) in feto-maternal tissues and increased the level of SCFAs in the amniotic fluid (25). Interestingly, the frequency of Breg was also increased in the intestine and femurs of fetuses. So the maternal microbiota shaped by prebiotic supplementation programs the offspring’s IS toward tolerance *in utero*. Remarkably, at 6 weeks of age, we still observe tolerance biomarkers in pups antenatally exposed to prebiotics, including an increase of Treg and Breg associated with an increased IL-10 secretion, and an increased Th1 and Th2 response. We have already demonstrated that *in utero* the consumption of prebiotics promotes a pro-tolerogenic environment (25), and herein we demonstrate that this immune environment endures at least until 6 weeks of life. The increase in Breg numbers might be explained by the increased abundance of *Lactobacillus* in the fecal microbiota, as this family has been shown to promote the differentiation of Breg in the spleen and in the mesenteric lymph nodes (49). Similarly, the increase in Treg numbers might be explained by the increased relative abundance of *Bifidobacterium*, which were shown to induce the generation of Treg (50). Together, these data show that the immune imprinting acquired *in utero* following the maternal consumption of prebiotics during pregnancy lasts over time and is associated with the transmission of a microbial signature.

We showed that this tolerogenic environment promoted by prebiotic supplementation mitigated FA development in offspring. Indeed, we observed a significant decrease in allergic symptoms associated with a decrease of allergic parameters (Th2 and wheat-specific IgG1) and an increase of tolerogenic parameters (wheat-specific IgA, Treg and Breg numbers, and TGF-β concentration). Previously, maternal supplementation of mice with prebiotics (scGOS/lcFOS) during pregnancy also reduced asthmatic symptoms in offspring, which was associated with an increase in Treg and higher concentrations of allergen-specific IgG2a in plasma (26). A protective effect of FOS supplementation during pregnancy was also demonstrated on atopic dermatitis-like skin lesions in a mouse model associated with lower serum concentrations of total IgG1 (28).

In our model, FA mice antenatally exposed to prebiotics display an increase in Actinobacteria, Prevotellaceae, and Lactobacillaceae in their stools. Interestingly, xylooligosaccharide supplementation in mice promoted intestinal *Prevotella* colonization, which was associated with a reduction of oxazolone-induced atopic dermatitis. In a maternal cohort, gastrointestinal presence of *Prevotella* spp. during pregnancy was associated with FA protection in children (51). Finally, the increased presence of Lactobacillaceae is of interest, as Lactobacillaceae are the most studied probiotics in the field of infancy supplementation to prevent or treat FA (52). Prenatal and postnatal administration of high doses of *Lactobacillus rhamnosus-GG* seems to be an effective approach to reduce the total IgE levels and atopic sensitization (53). Surprisingly, in our study, the modulation of the microbiota in FA mice was not associated with a modulation of metabolite concentration in the feces nor in the colon (data not shown). These data suggest that the prevention of FA may not be mediated by the metabolites but by a direct effect of the microbiota or the pro-tolerogenic environment setup in early life. In conclusion, GOS/inulin supplementation during pregnancy increases the abundance of FA protective strains in the offspring’s gut microbiota, which might explain the protective effect observed in our study.

In summary, our findings show, for the first time, that prebiotic supplementation exclusively during pregnancy is an effective strategy to mitigate FA in offspring by acting on microbial and immune tolerance mechanisms. We are currently investigating the effects of such a strategy in humans in a randomized clinical trial (PREGRALL) that seeks to demonstrate the effects of supplementation with prebiotics during pregnancy on the occurrence of atopic dermatitis in children (54). These results will help, in the future, to set up dietary recommendations relating to prebiotic consumption for pregnant women at risk of having an allergic child.

## Data Availability Statement

The datasets presented in this study can be found in online repositories. The names of the repository/repositories and accession number(s) can be found below: https://www.ncbi.nlm.nih.gov/; PRJNA751386.

## Ethics Statement

The animal study was reviewed and approved by Ethics Committee on Animal Experimentation of the Pays de la Loire region.

## Author Contributions

Study conceptualization: AS, GB, CB, and MB. Methodology: AS, GB, CB, and MB. Formal analysis: AS, CB, MB, CC, and MM. Investigation: AS, CB, CC, AR, AD, and AB. Resources: MB and GB. Writing (original draft preparation): AS and CB. Writing (review and editing): CB, AS, WD, MB, GB, SB, and CC. Supervision: MB. Project administration: MB. Funding acquisition: MB. All authors contributed to the article and approved the submitted version.

## Funding

This study was supported by the “Région Pays de la Loire” through the RFI Food for Tomorrow/CAP ALIMENT (COPADAF project), the ANR through the CIMMAP project and the SFA and SFN grants.

## Conflict of Interest

The authors declare that the research was conducted in the absence of any commercial or financial relationships that could be construed as a potential conflict of interest.

## Publisher’s Note

All claims expressed in this article are solely those of the authors and do not necessarily represent those of their affiliated organizations, or those of the publisher, the editors and the reviewers. Any product that may be evaluated in this article, or claim that may be made by its manufacturer, is not guaranteed or endorsed by the publisher.

## Supplementary Material

Supplementary Figure 1 Prebiotics supplementation does not modulate the alpha diversity. Analysis was based on 16S rDNA sequencing (region V3-V4) of observed species richness as indicators of α-diversity at **(A)** day 0; D0, **(B)** day 18 of gestation; D18P, **(C)** day 10 of lactation; D10L, **(D)** day 21 of lactation; D21L. α-diversity data are displayed as mean ± S.E.M; P-values were determined using one-way ANOVA or Mann-Whitney test. **(E)** Analyze of indices for species evenness at D0, D18P, D10L, D21L (Shannon index). **(F)** Graphical representation and heatmap of operational taxonomic unit (OTU) variance in stools of controls and prebiotics supplemented mothers stools D21L.

Supplementary Figure 2 Gating strategy used for flow cytometry analysis. **(A)** Gating strategy used after immunostaining to evaluate **(A)** all T cell subsets and **(B)** all B cell subsets.

Supplementary Figure 3 Frequency of T and B cell subsets in the mesenteric and inguinal lymph nodes of CTL and PB offspring at 6 weeks of age. Frequencies of **(A)** CD3^+^CD4^+^CD25^+^FoxP3^+^, **(B)** CD3^+^CD8^+^FoxP3^+^, **(C)** CD19^+^CD9^+^, **(D)** CD19^+^CD25^+^, **(E)** CD3^+^CD4^+^IL-4^+^ and **(F)** CD3^+^CD4^+^IFN-γ^+^ cells in mesenteric (for T cells) and inguinal (for B cells) lymph nodes. All data are displayed as the mean ± standard error (n=8 to 12 animals per group); p-values were determined using the Mann-Whitney test (*p < 0.05, **p < 0.01).

Supplementary Figure 4 Frequency of T and B cell subsets in the mesenteric and inguinal lymph nodes of CTL-FA and PB-FA offspring at 6 weeks of age. Frequencies of **(A)** CD3^+^CD4^+^CD25^+^FoxP3^+^, **(B)** CD3^+^CD8^+^FoxP3^+^, **(C)** CD19^+^CD9^+^, **(D)** CD19^+^CD25^+^, **(E)** CD3^+^CD4^+^IL-4^+^ and (F) CD3^+^CD4^+^IFN-γ^+^ cells in mesenteric (for T cells) and inguinal (for B cells) lymph nodes. All data are displayed as the mean ± standard error (n=8 to 12 animals per group); p-values were determined using the Mann-Whitney test (*p < 0.05, **p < 0.01).
