# Supplementary figures and images for: Prebiotic Supplementation During Gestation Induces a Tolerogenic Environment and a Protective Microbiota in Offspring Mitigating Food Allergy
Source: Front Immunol. 2022 Jan 5;12:745535. doi: 10.3389/fimmu.2021.745535 (PMC8769244; doi:10.3389/fimmu.2021.745535)

## Slide 1
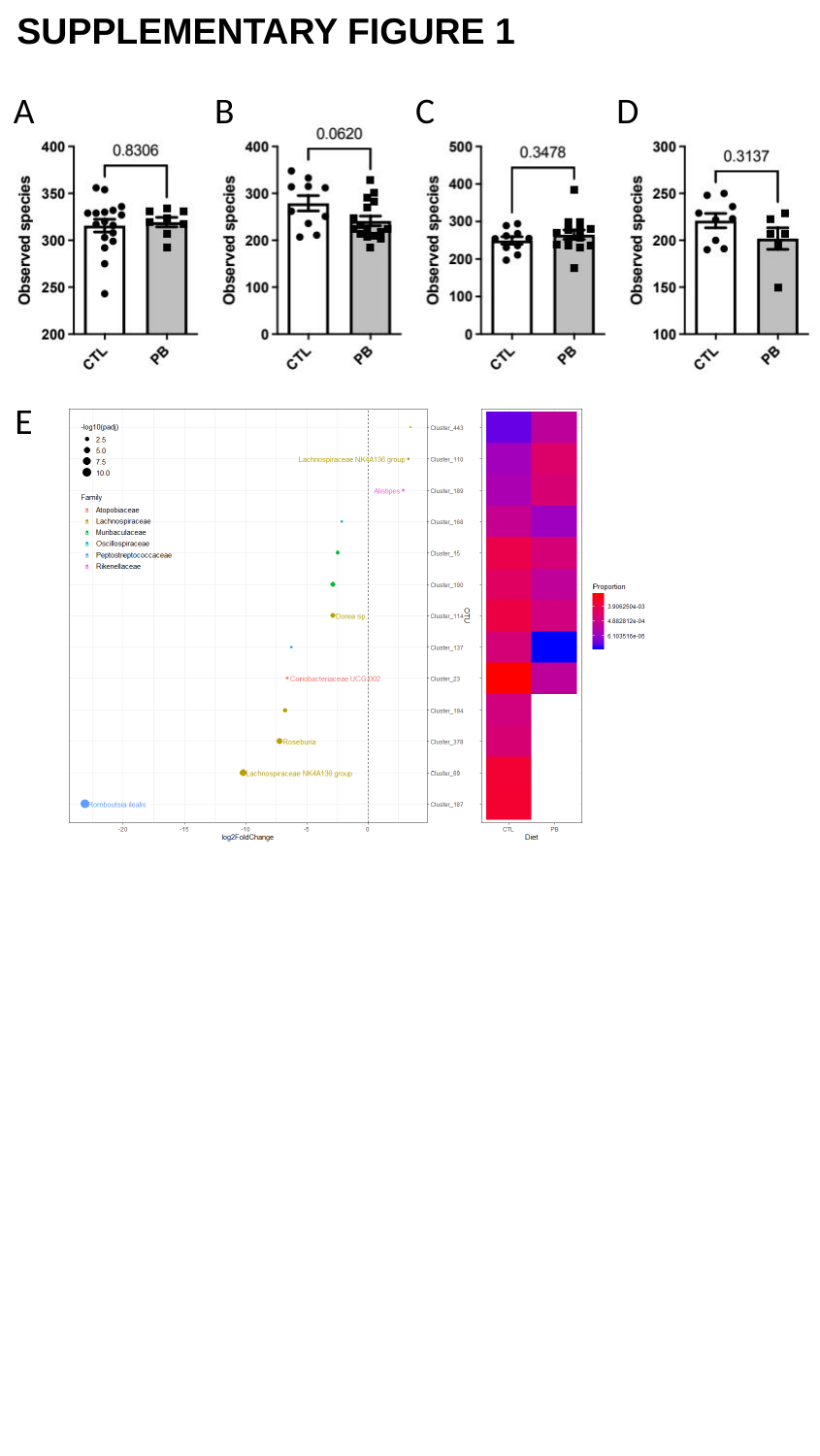

SUPPLEMENTARY FIGURE 1
A
B
C
D
E

Supplement: Supplementary Figure 1 — Prebiotics supplementation does not modulate the alpha diversity. Analysis was based on 16S rDNA sequencing (region V3-V4) of observed species richness as indicators of α-diversity at (A) day 0; D0, (B) day 18 of gestation; D18P, (C) day 10 of lactation; D10L, (D) day 21 of lactation; D21L. α-diversity data are displayed as mean ± S.E.M; P-values were determined using one-way ANOVA or Mann-Whitney test. (E) Analyze of indices for species evenness at D0, D18P, D10L, D21L (Shannon index). (F) Graphical representation and heatmap of operational taxonomic unit (OTU) variance in stools of controls and prebiotics supplemented mothers stools D21L. [file Presentation_1.ppt]

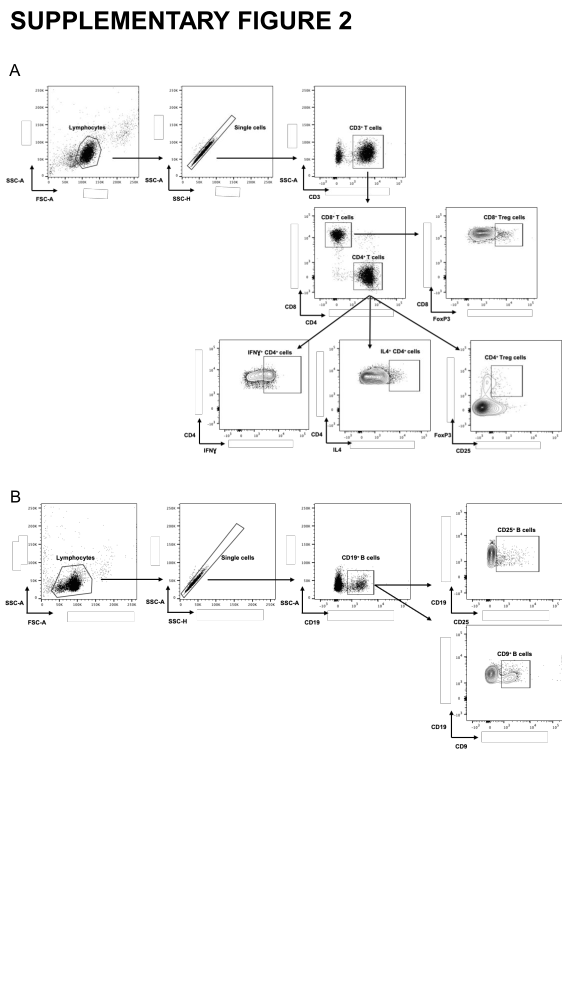

Supplement: Supplementary Figure 2 — Gating strategy used for flow cytometry analysis. (A) Gating strategy used after immunostaining to evaluate (A) all T cell subsets and (B) all B cell subsets. [file Image_1.tiff]

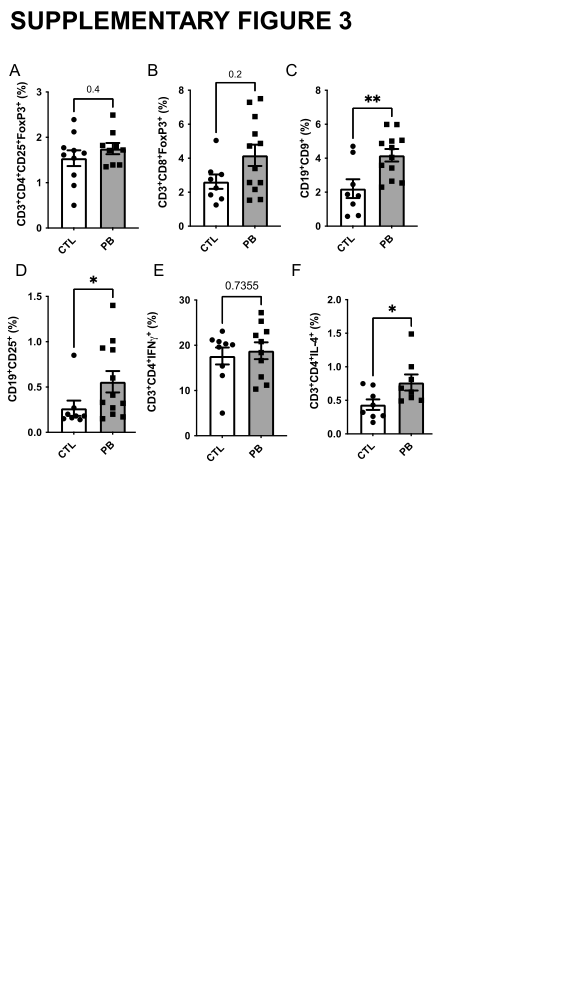

Supplement: Supplementary Figure 3 — Frequency of T and B cell subsets in the mesenteric and inguinal lymph nodes of CTL and PB offspring at 6 weeks of age. Frequencies of (A) CD3+CD4+CD25+FoxP3+, (B) CD3+CD8+FoxP3+, (C) CD19+CD9+, (D) CD19+CD25+, (E) CD3+CD4+IL-4+ and (F) CD3+CD4+IFN-γ+ cells in mesenteric (for T cells) and inguinal (for B cells) lymph nodes. All data are displayed as the mean ± standard error (n=8 to 12 animals per group); p-values were determined using the Mann-Whitney test (*p < 0.05, **p < 0.01). [file Image_2.tiff]

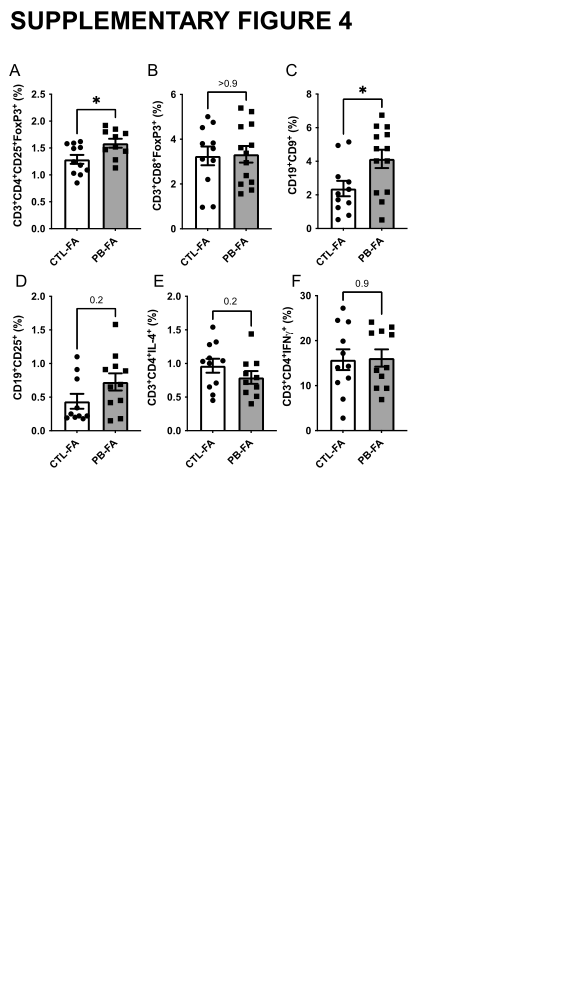

Supplement: Supplementary Figure 4 — Frequency of T and B cell subsets in the mesenteric and inguinal lymph nodes of CTL-FA and PB-FA offspring at 6 weeks of age. Frequencies of (A) CD3+CD4+CD25+FoxP3+, (B) CD3+CD8+FoxP3+, (C) CD19+CD9+, (D) CD19+CD25+, (E) CD3+CD4+IL-4+ and (F) CD3+CD4+IFN-γ+ cells in mesenteric (for T cells) and inguinal (for B cells) lymph nodes. All data are displayed as the mean ± standard error (n=8 to 12 animals per group); p-values were determined using the Mann-Whitney test (*p < 0.05, **p < 0.01). [file Image_3.tiff]
